# Prediction model for major bleeding in anticoagulated patients with cancer-associated venous thromboembolism using machine learning and natural language processing

**DOI:** 10.1007/s12094-024-03586-2

**Published:** 2024-09-14

**Authors:** Andrés J. Muñoz Martín, Ramón Lecumberri, Juan Carlos Souto, Berta Obispo, Antonio Sanchez, Jorge Aparicio, Cristina Aguayo, David Gutierrez, Andrés García Palomo, Diego Benavent, Miren Taberna, María Carmen Viñuela-Benéitez, Daniel Arumi, Miguel Ángel Hernández-Presa

**Affiliations:** 1 Medical Oncology Service, Hospital General Universitario Gregorio Marañón Universidad Complutense, Madrid, Spain; 2 Hematology Service, Clínica Universidad de Navarra, Pamplona, Spain; 3 CIBERCV, Carlos III Health Institute, Madrid, Spain; 4 Hematology Department, Santa Creu i Sant Pau Hospital, Barcelona, Spain; 5 Oncology Department, Infanta Leonor Hospital, Madrid, Spain; 6 Oncology Department, Puerta de Hierro Hospital, Madrid, Spain; 7 Oncology Department, Polytechnic and University Hospital of La Fé, Valencia, Spain; 8 Oncology Department, Infanta Sofía Hospital, Madrid, Spain; 9 Oncology Department, Fuenlabrada Hospital, Madrid, Spain; 10 Oncology Department, University Hospital of León, León, Spain; 11 Savana Research, Madrid, Spain; 12 Medicine Department, Facultad de Medicina, Universidad Complutense, Madrid, Spain; 13 Pfizer S.L.U. Medical Department, Madrid, Spain

**Keywords:** Venous thromboembolism, Machine learning, Natural language processing, Anticoagulants, Cancer, Major bleeding

## Abstract

**Purpose:**

We developed a predictive model to assess the risk of major bleeding (MB) within 6 months of primary venous thromboembolism (VTE) in cancer patients receiving anticoagulant treatment. We also sought to describe the prevalence and incidence of VTE in cancer patients, and to describe clinical characteristics at baseline and bleeding events during follow-up in patients receiving anticoagulants.

**Methods:**

This observational, retrospective, and multicenter study used natural language processing and machine learning (ML), to analyze unstructured clinical data from electronic health records from nine Spanish hospitals between 2014 and 2018. All adult cancer patients with VTE receiving anticoagulants were included. Both clinically- and ML-driven feature selection was performed to identify MB predictors. Logistic regression (LR), decision tree (DT), and random forest (RF) algorithms were used to train predictive models, which were validated in a hold-out dataset and compared to the previously developed CAT-BLEED score.

**Results:**

Of the 2,893,108 cancer patients screened, in-hospital VTE prevalence was 5.8% and the annual incidence ranged from 2.7 to 3.9%. We identified 21,227 patients with active cancer and VTE receiving anticoagulants (53.9% men, median age of 70 years). MB events after VTE diagnosis occurred in 10.9% of patients within the first six months. MB predictors included: hemoglobin, metastasis, age, platelets, leukocytes, and serum creatinine. The LR, DT, and RF models had AUC-ROC (95% confidence interval) values of 0.60 (0.55, 0.65), 0.60 (0.55, 0.65), and 0.61 (0.56, 0.66), respectively. These models outperformed the CAT-BLEED score with values of 0.53 (0.48, 0.59).

**Conclusions:**

Our study shows encouraging results in identifying anticoagulated patients with cancer-associated VTE who are at high risk of MB.

**Supplementary Information:**

The online version contains supplementary material available at 10.1007/s12094-024-03586-2.

## Introduction

Venous thromboembolism (VTE), which includes deep vein thrombosis and pulmonary embolism, is a major complication and a leading cause of death in cancer patients [1]. The annual incidence of VTE is 3–15% in active cancer patients depending on the cancer type and location [2, 3]. In these patients, anticoagulant treatment increases bleeding and medical complications [4, 5]. In this regard, the American Society of Clinical Oncology (ASCO) guidelines recommend low-molecular weight heparin (LMWH) or direct oral anticoagulants (DOACs) as a first-line treatment for cancer-related VTE in hospitalized patients without bleeding. LMWH or DOACs are preferred over vitamin K antagonists (VKA) because of higher efficacy. Anticoagulant therapy is recommended for at least 6 months and extended treatment is suggested for active cancer patients or patients receiving anti-cancer therapy [6].

Researchers have reported the cumulative incidence of major bleeding (MB) events among cancer patients receiving anticoagulants as 5.9% and 8.7% at three and six months after treatment onset, respectively, compared with 2.6% and 4.2% in non-anticoagulated cancer patients [7]. Consequently, an individualized assessment of VTE recurrence and bleeding risk should be made in cancer patients with VTE before starting anticoagulant treatment to personalize anticoagulant-specific therapies, especially when considering prolonging treatment beyond the first six months following the initial VTE event [8].

Several studies have explored the risk of bleeding events in cancer patients receiving anticoagulant treatment [9–11]. However, existing predictive models and validated scores to assess bleeding risk in this population have limitations in methodology and accuracy. For example, the CAT-BLEED score, derived from the Hokusai VTE Cancer clinical trial [12], might not be generalizable to broader populations and displays poor to moderate predictive performance in its internal validation.

Analyzing real-world data (RWD) can help identify risk factors for bleeding events in patients with cancer-related VTE receiving anticoagulant treatment. Recent studies have extracted and analyzed unstructured clinical information from patients’ electronic health records (EHRs) to describe patients’ clinical characteristics, disease management, and predictive factors for disease prognosis [13–15]. Moreover, using natural language processing (NLP) and machine learning (ML), our group recently developed a predictive model for six-month VTE recurrence among anticoagulated cancer patients within the larger PredictAI study [13].

To expand upon our previous findings in cancer patients with VTE, in this study we aimed to develop a predictive model for MB in anticoagulated cancer patients within the first six months post-VTE diagnosis using NLP and ML [13–15] to analyze the unstructured, free-text narratives in patients’ EHRs from nine Spanish hospitals. Secondary objectives were to describe the prevalence and incidence of VTE in cancer patients receiving anticoagulants, their baseline clinical characteristics, and bleeding events during follow-up.

## Methods

### Study design and population

PredictAI was an observational, retrospective, and multicenter study that analyzed unstructured clinical information in patients' EHRs. We included all anticoagulation-treated adult patients (aged 18 or older) with active cancer and VTE from January 1, 2014, to December 31, 2018. Additional inclusion and exclusion criteria, a priori sample size calculations, and definitions of active cancer, VTE and VTE diagnosis are included in the Supplementary Information methods.

**Fig. 1:**
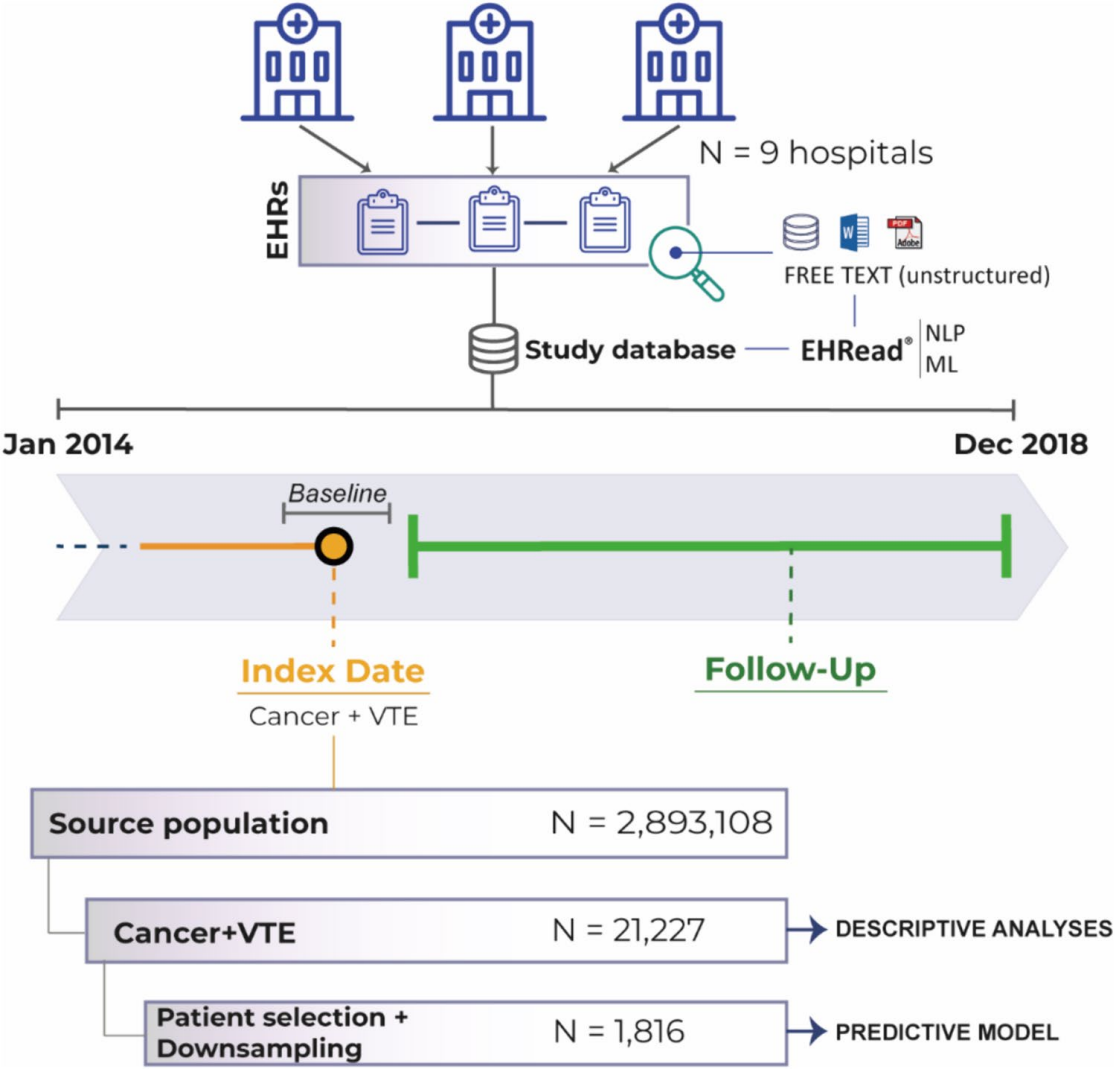
Study design and sample Using the EHRead^®^ technology, the unstructured clinical data from patients’ EHRs were extracted and analyzed at two different time windows, namely index date and follow-up (see “Methods” for details). From a source population of 2,893,108 individuals, a total of 21,227 patients diagnosed with cancer and VTE were identified. *ML* machine learning, *NLP* natural language processing, *VTE* venous thromboembolism

A cross-sectional analysis was conducted on all patients at the time of inclusion, hereafter referred to as the index date, the timepoint when either active cancer or VTE first appeared in the EHRs during the study period (Fig. 1). Incidence and prevalence calculations were based on the total number of cancer patients at risk halfway through the study period. The follow-up period was from the index date until the end of the study period or until the last EHR was available. A subset of patients with available robust data for predictive analysis was developed.

### Data source and study variables

The unstructured clinical information from EHRs was extracted from nine third-level representative hospitals within the Spanish National Healthcare Network (refer to Supplementary Information methods). Data were collected from various reports, such as radiology reports, outpatient clinic reports, hospitalization reports, hospital discharge summaries, emergency room treatment notes, and prescriptions, in all available departments in each participating site, including inpatient hospital, outpatient hospital, and emergency room.

Study variables were analyzed as part of a curation process to ensure data quality and integrity. Sociodemographic and clinical variables were collected, including diagnoses, medications, and treatment history. Bleeding events [i.e., MB and clinically relevant non-major bleeding (CRNMB)] were defined using International Society on Thrombosis and Haemostasis (ISTH) criteria [16]. MB was defined as a hemoglobin decrease of 2 g/dL or more within one week and transfusion of at least two red blood cell units. Other definitions used are described in the Supplemental Information methods. Patients treated with any type of anticoagulants were included with a detailed breakdown for VKA and heparin (i.e., unfractionated heparin and LMWH) treatments.

### Extraction of the unstructured information from EHRs

We used *EHRead*^*®*^ technology [14, 15] to extract unstructured data from de-identified EHRs using NLP and ML and translating it into a study database.

A statistical model was generated to describe the study population’s demographic and clinical characteristics. The terminology considered by this technology included codes, synonyms, and definitions used in clinical documentation and was based on Systematized Nomenclature of Medicine Clinical Terms (SNOMED CT) [17]. An initial data quality assessment was conducted to evaluate EHRs and patient distribution across hospital departments and years during the study period (Fig. S1). Additional information about *EHRead*^*®*^ technology is in the Supplemental Information methods.

### Evaluation of EHRead^®^’s performance

*EHRead*^*®*^’ was externally evaluated for its ability to identify patient records containing key variables related to cancer and VTE as previously published [15] (see Supplemental Information methods for details). The evaluation compared the reading output and a gold standard corpus of medical records annotated by expert physicians in each participating hospital. The result of this comparison is expressed in terms of the standard metrics of precision (positive predictive value or PPV), recall (sensitivity), and their harmonic mean, F1-score. The F1score for most variables was ≥ 80% and are included in Table S1 [13].

### Statistical analyses

#### Descriptive analyses

From eligible patients within the study period we selected all available EHRs prior to the study end to help build a more complete picture of each patient’s baseline data such as comorbidities. Baseline data regarding pharmacological treatments and laboratory parameters were analyzed using 6-month windows around index date. Follow-up analyses for bleeding events were performed for the entire duration of the follow-up period and for the 6 months post-baseline, independently. We used frequency tables to describe categorical variables and summary tables for numerical variables that include the mean, standard deviation (SD), median, first quartile (Q1), and third quartile (Q3). The absence of information in patients’ EHRs was considered a ‘true zero’ for binary variables and missing data for numerical variables. Methods for calculating in-hospital prevalence and annual incidence rates of VTE in cancer patients are included in Supplementary Information methods.

#### Predictive model for MB events

A clinical predictive model for the occurrence of MB within six months after a VTE diagnosis was developed in patients with (a) no history of MB prior to VTE diagnosis date, (b) in whom the index date coincided with VTE diagnosis date (i.e., VTE diagnosis occurs after cancer diagnosis), and (c) with a minimum available follow-up of 6 months (for patients with no MB event). Patients with MB within six months were considered the positive class, whereas patients with no MB within six months were labeled as the negative class. We used three types of models, mainly logistic regression (LR), decision tree (DT) and random forest (RF), which were trained and evaluated for their performance metrics. The development of the model included: (1) population selection to prevent biases and class imbalance; (2) clinically- and ML-driven feature selection on an initial set of clinically relevant variables; (3) model training and validation. Lastly, model selection evaluation was based on performance metrics, namely area under the receiver operating characteristic curve (ROC-AUC), precision (PPV), recall (sensitivity), accuracy, and F1-score. An external validation of the CAT-BLEED score was performed to compare the performance of our newly developed model with previously published scores. Additional information regarding the construction of the predictive model is included in the Supplementary Information methods.

## Results

### Population definition and descriptive analyses

EHRs from 2,893,108 patients were processed from 9 hospitals during the study period (Fig. 1). The estimated in-hospital prevalence of cancer-related VTE across the study period was 5.8%, and the annual incidence was (2.7–3.9%) (Table 1). The study population comprised 21,227 patients with active cancer and VTE who underwent anticoagulant treatment.

**Table 1:** Estimated in-hospital prevalence of cancer-related VTE

	n (%)
Period prevalence 2014–2018^a^	9352 (5.8)
Annual incidence	
2014	1470 (2.7)
2015	3528 (3.9)
2016	4093 (3.4)
2017	3803 (3.3)
2018	2775 (3.8)

For the estimation of yearly incidences, only data from sites with available records for the year of interest were analyzed

^a^Calculation based on the total number of patients at risk (with cancer) at midpoint of study period (N = 160,049)

Approximately half of the patients were male (53.9%; n = 11,431) with a median age (Q1, Q3) of 70 (59, 80) years at baseline (Table 2). The VTE- and cancer-related clinical characteristics at baseline are shown in Table 2. The most common type of VTE was deep vein thrombosis (68.2% of patients; n = 14,471), followed by pulmonary embolism (28.4%; n = 6038). The most common cancer locations at baseline were colorectal (10.1% of patients, n = 2143), lung (8.5%, n = 1810), and hematologic (7.3%, n = 1547). Overall, 16.9% (n = 3584) had tumors with mucosal involvement, and 44.6% (n = 9466) of patients had metastasis. The most common comorbidities at baseline were hypertension (59.2%; n = 12,565), medical conditions of neurological nature (52.5%; n = 11,138), and dyslipidemia (41.1%; n = 8715) (Table 3).

**Table 2:** Demographic and clinical characteristics at baseline

	N = 21,227
Demographics	
Sex	
Male	11,431 (53.9)
Female	9796 (46.1)
Age at index date (years)	
Mean (SD)	68.54 (14.98)
Median (Q1–Q3)	70 (59, 80)
Clinical characteristics	
VTE-related characteristics	
VTE subtype n (%)	
Deep vein thrombosis	14,471 (68.2)
Pulmonary embolism	6038 (28.4)
Synchronic pulmonary and deep vein thrombosis	1300 (6.1)
Visceral vein thrombosis	1724 (8.1)
Splenic thrombosis	898 (4.2)
Portal thrombosis	812 (3.8)
Other type of VTE	347 (1.6)
Cancer-related characteristics	
Primary cancer location n (%)	
Colorectal cancer	2143 (10.1)
Lung cancer	1810 (8.5)
Hematologic cancer	1547 (7.3)
Bladder cancer	1502 (7.1)
Breast cancer	1395 (6.6)
Prostate cancer	1191 (5.6)
Pancreatic cancer	1053 (5)
Endometrial cancer	818 (3.9)
Cervix cancer	570 (2.7)
Gastric cancer	423 (2)
Brain cancer	421 (2)
Esophageal cancer	376 (1.8)
Kidney cancer	299 (1.4)
Bile duct cancer	222 (1)
Ovarian cancer	187 (0.9)
Thyroid cancer	155 (0.7)
Vulvar cancer	12 (0.1)
Vaginal cancer	2 (0)
Other cancer locations	7405 (34.9)
Age at cancer diagnosis (years)	
Mean (SD)	65.54 (15.46)
Median (Q1, Q3)	67 (56, 77)
Other characteristics n (%)	
Complete remission n (%)	1054 (5)
Metastases	9466 (44.6)
Tumors with mucosal involvement	3584 (16.9)

Included population comprised all AC-treated adult patients with a diagnosis of both active cancer and VTE

**Table 3:** Comorbidities and procedures at baseline

	N = 21,227
Comorbidities n (%)	
Hypertension	12,565 (59.2)
Neurological diseases^a^	11,138 (52.5)
Dyslipidemia	8715 (41.1)
Psychiatric disorders	6855 (32.3)
Diabetes mellitus	6200 (29.2)
Heart failure	5284 (24.9)
Kidney failure	5061 (23.8)
Atrial fibrillation	3972 (18.7)
Cerebrovascular accident (CVA)	3823 (18)
Chronic obstructive pulmonary disease (COPD)	3478 (16.4)
Varicose veins	3430 (16.2)
Arterial thromboembolic events (ATEs)	2634 (12.4)
Peripheral artery disease	1477 (7)
Acute myocardial infarction	1436 (6.8)
Inherited thrombophilia	1233 (5.8)
Asthma	962 (4.5)
Chronic hepatopathy	781 (3.7)
Transient ischemic attack (TIA)	617 (2.9)
Procedures	
Central venous catheter	1170 (5.5)
Port-a-cath	1060 (5)
Peripherally inserted central catheter (PICC)	447 (2.1)
Hickman catheter	41 (0.2)
Other catheter	1017 (4.8)

Included population comprised all AC-treated adult patients with a diagnosis of both active cancer and VTE

^a^Neurological diseases include peripheral neuropathy

The medication patterns and laboratory values at baseline are displayed in Table S2 and Table S3, respectively. Regarding anticoagulant treatment, most patients were prescribed LMWH (84%; n = 17,830) and/or VKA (22.2%; n = 4710). As for chemotherapy, 8.2% (n = 1747) were using platinum compounds, followed by taxanes (5.1%; n = 1088). The most prescribed monoclonal antibody agents at baseline were panitumumab (3.7%; n = 790) and bevacizumab (2.2%; n = 461). The most prescribed tyrosine kinase inhibitor was sorafenib (0.4%; n = 86) (Table S2).

**Fig. 2:**
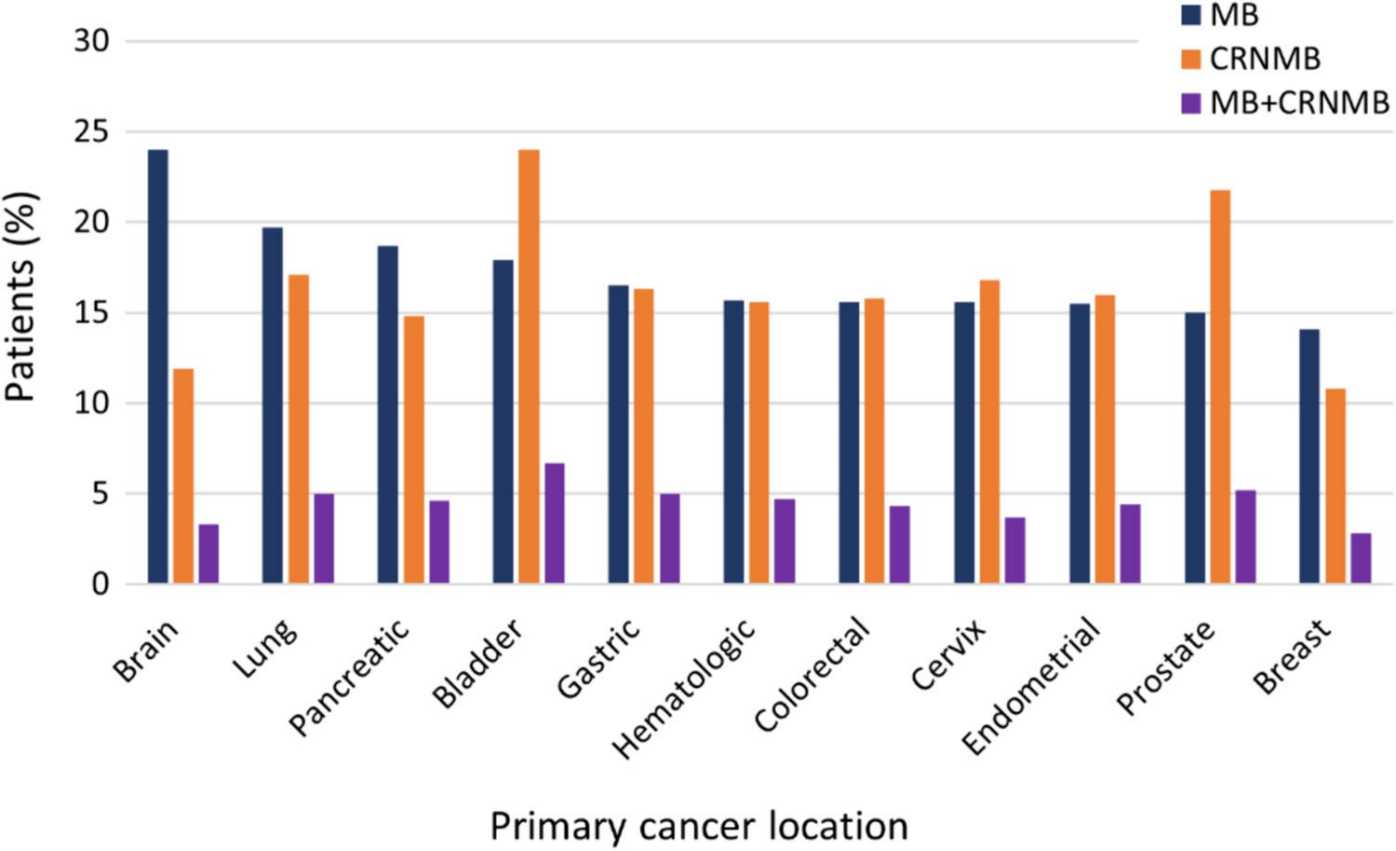
MB and CRNMB events by primary cancer location during follow-up Vertical bars depict the number of patients suffering at least from either one MB event (blue), a CRNMB (orange) event, or both MB and CRNMB (purple) during follow-up across the most common primary cancer locations (according to ISTH criteria). Please note that the MB + CRNMB group is not a direct sum of MB and CRNMB but represent patients who had suffered both types of events during the follow-up period. *CRNMB* clinically relevant non-major bleeding, *MB* major bleeding

The median (Q1, Q3) duration of follow-up across patients was 8.4 (1.32, 24.4) months. During the entire follow-up period, at least one MB or CRNMB event was registered in 16.5% (n = 3497) and 16.3% (n = 3457) of patients respectively (Table S4). Figure 2 shows the distribution of MB and CRNMB events across the most common primary cancer locations during the entire follow-up period. Among these, brain cancer was associated with higher incidence of MB events (24% of patients within the location subgroup; n = 101) followed by esophageal cancer (19.9%, n = 75) and lung (19.7%; n = 357). On the other hand, CRNMB events were more frequent in patients with bladder cancer (24%; n = 360) and prostate cancer (21.8%; n = 260). Table S4 shows bleeding rates across all primary cancer locations analyzed in the study.

### Predictive model development and validation

**Fig. 3:**
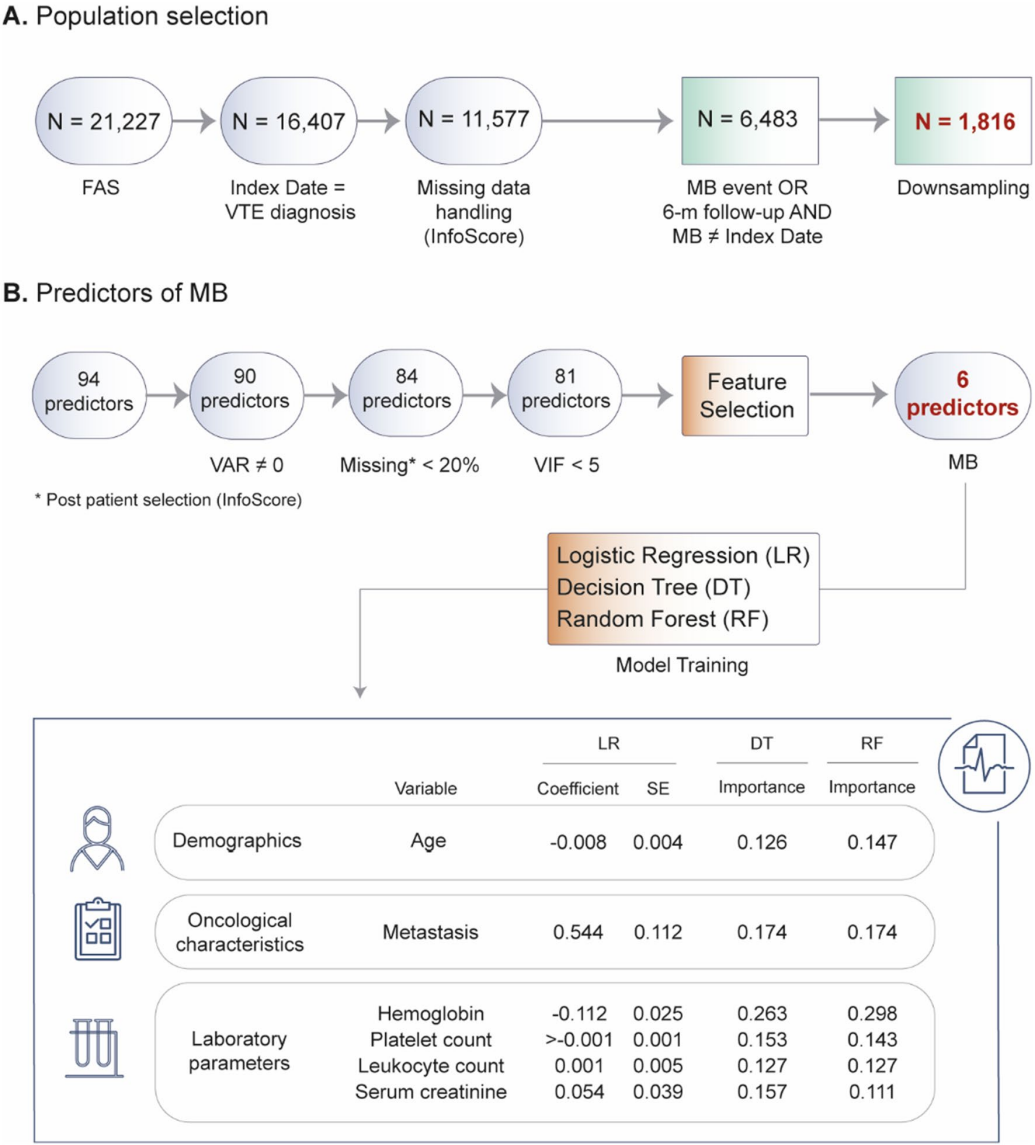
Predictive model for MB in oncology patients with VTE. **A** Diagram depicting the population selection flow for the prediction model. From an initial included population of 21,227 patients, the set used to build predictive model comprised 1816 patients after applying additional inclusion criteria, patient selection via InfoScore, and downsampling techniques (see Supplementary Methods for details). **B** Diagram showing the selection of predictors (i.e., independent variables). From a starting set of 94 variables, 81 predictors were obtained after excluding variables with zero variance, numeric variables with missing values over 20%, and collinear variables. The feature selection identified 6 predicting factors for MB events within the first six months following VTE, namely patient’s age, presence of metastasis, hemoglobin levels, platelet count, leukocyte count, and serum creatinine levels. The relative importance of each variable is shown. *MB* major bleeding (according to ISTH criteria), *ROC-AUC* receiver operating characteristic-area under the curve, *VAR* variance, *VIF* variance inflation factor (see Supplementary Information methods)

Among the 16,407 cancer patients with VTE diagnosis that were diagnosed at index date (77.3% of the total), 10.9% (n = 1790) had at least one MB event within the first six months after VTE diagnosis. To build the model, its population selection first yielded a balanced dataset of 1816 patients (Fig. 3a). Of these, 1348 patients (75%) were used for training the predictive models and 450 (25%) for their internal validation. Initial set of variables (predictors) considered for the development of the predictive model for MB is shown in Table S5. The feature selection process yielded a total of six variables, including patient’s age, presence of metastasis, and the following laboratory parameters at VTE diagnosis date: hemoglobin, platelet count, leukocyte count, and serum creatinine. Data distribution for the six key predictors in the training vs. testing patient sets is shown in Table S6. Figure 3 shows the coefficients and feature importance obtained for each predictor on LR, DT, and RF models, respectively. Model performance metrics and ROC and calibration curves are shown in Table S7 and Figs. S2 and S3, respectively, including an external validation of the CAT-BLEED score using our study data. The LR model had an AUC-ROC (95% confidence interval, CI) of 0.60 (0.55, 0.65), the DT of 0.60 (0.55, 0.65) and the RF of 0.61 (0.56, 0.66). CAT-BLEED had an AUC-ROC (95% CI) of 0.53 (0.48, 0.59). Figure S4 shows the graphical representation of the DT classifier model.

## Discussion

Using our ML predictive model we estimated a 5.8% prevalence of VTE in hospitalized cancer patients receiving anticoagulant therapy and an annual incidence of MB events after VTE diagnosis ranging from 2.7 to 3.9%, which is consistent with previous reports [18]. Our main result is the development of a ML model for the prediction of MB events in cancer patients treated with anticoagulation therapy within the 6 months following VTE diagnosis. MB predictors included hemoglobin levels, presence of metastasis, patient's age, platelet count, leukocyte count, and serum creatinine levels at baseline. The predictive performance on the validation set was moderate [ROC-AUC of 0.61 (0.56, 0.66)], outperforming previously developed risk scores with low to moderate predictive performance when applied to our data [ROC-AUC of 0.53 (0.48, 0.59) for CAT-BLEED]. These findings suggest this newly developed model has the potential to improve the prevention and clinical management of bleeding in this population.

Cancer location is a major risk factor for recurring VTE and bleeding risk [19, 20], with the highest risk of VTE associated with pancreas, uterus, lung, stomach, kidney, and brain cancer [21]. The most common cancer locations in our cohort of 21,227 patients were colorectal (10.1%) and lung (8.5%), followed by hematologic and bladder cancer (both 7%), which is in line with previous studies [10, 22, 23]. In the randomized, open-label Hokusai study including 1046 patients, colorectal (15%), lung (14%), and breast (11.5–12.3%) were also the most common cancer locations across groups. These rates also match those reported in the Caravaggio trial in patients with cancer and VTE [10, 22], as well as the rates found in the TESEO prospective registry of cancer patients in Spain [23].

We used our advanced NLP and ML methods to accurately detect bleeding events, often difficult to find in patient EHRs because the unstructured character of this information [24]. During the entire follow-up period (with a median of 8.4 months), 16.5% of all patients at least had one MB event. These rates were reduced to 10.9% when calculated during only the first 6 months of follow-up, similar to a previous study using RWE [7]. MB events during 3-month anticoagulation treatment period in registries or during a 6-month period in clinical trials were documented in 3.1–7.4% [10, 19, 20, 22]. The higher incidence reported here compared to clinical trials may be due to our longer follow-up time, differences in the definition of MB, methodology, and wider inclusion/exclusion criteria.

Our methodology identified primary predictors associated with MB, which are similar to findings from other studies in cancer patients with VTE [20, 25, 26]. Our ML approach on a much larger series of real-world patients partially replicated their results by identifying metastasis, creatinine levels, and hemoglobin levels as predictors of MB, in addition to patient’s age, platelet count, and leukocyte count. Notably, the PredictAI LR model found that lower hemoglobin and platelet levels and higher leukocyte and creatinine levels were associated with increased VTE risk. In this regard, although elevated leukocyte counts in cancer patients with acute VTE has been previously related with a higher incidence of VTE recurrences, this interesting finding has been scarcely reported [20, 27].

Our study has important strengths. The predictive model shows promising results in identifying patients with cancer and VTE who are at a high risk for MB compared to previous models that have shown suboptimal performance metrics and limited value in clinical practice [12, 23, 28]. Furthermore, performance of the CAT-BLEED model was poor when applied to our data maybe due to our model relying on the inclusion of a larger and more representative series of real-world patients. Then, our results present the potential to improve medical management and prevent future bleeding complications. Ongoing steps involve an independent external validation of the model using a patient cohort from the TESEO registry of thrombosis and cancer (Spanish Society of Medical Oncology) and the subsequent development of a clinical score for its easy application in healthcare settings.

Despite our findings, this study has some limitations. Our results relied on the availability and accuracy of EHRs; therefore, not all variables included in the study were available in all records, which could potentially affect the robustness of our predictive model. In addition, DOACs were not reported due to their expected limited use during the study period. The first clinical evidence regarding the efficacy of DOACs in cancer patients was published in December 2017 [19] and data from the TESEO registry promoted by the Spanish Society of Medical Oncology showed only 0.6% of patients took DOACs between 2018 and 2019 [23]. Therefore, this could affect the generalizability of the findings, especially in current clinical settings where DOACs are more widely utilized. Another limitation is that the definition and accurate detection of MB in patients’ EHRs was exclusively based on the ISTH criteria, which had to be adapted to the SNOMED CT terminology used by the NLP system. Moreover, CRNMB bleeding events in this category were not easily detected in the patients’ EHRs, which could explain why the detection rates of CRNMB events were lower than expected. Another limitation is that the internal validation was carried out by a filtered group of patients, in which the case/non-case ratio was 1:1. Therefore, the resulting metrics should not be extrapolated to the entire population of cancer patients with VTE. Finally, the model may be subject to survival bias as the most critical and the healthiest patients may have been excluded from the training set due to a lack of robust follow-up.

To the best of our knowledge, PredictAI is the first study to use NLP to extract unstructured information from EHRs in a multicenter setting and use it to build a predictive model for MB in cancer patients with VTE. Physicians should assess bleeding risk when considering anticoagulant therapy after VTE in neoplastic patients, particularly in elderly individuals with metastasis, altered blood counts, and/or renal function, and adjust treatment strategies accordingly. Our model demonstrates encouraging outcomes in identifying cancer-associated VTE patients who are at a higher risk of MB, outperforming previously developed clinical scores.

## Supplementary Information

Below is the link to the electronic supplementary material.

Supplementary file1 (DOCX 975 KB)

## Data Availability

Data are available on reasonable request to the sponsor that will assess the proposal, and, if applicable, proceed to the data sharing.

## Abbreviations

ASCO: American Society of Clinical Oncology
CRNMB: Clinically relevant non-major bleeding
DT: Decision tree
DOAC: Direct oral anticoagulant
EHR: Electronic health record
ISTH: International Society on Thrombosis and Haemostasis
LMWH: Low molecular weight heparin
LR: Logistic regression
MB: Major bleeding
ML: Machine learning
NLP: Natural language processing
RF: Random forest
RWD: Real world data
SNOMED CT: Systematized Nomenclature of Medicine Clinical Terms
VTE: Venous thromboembolism

## Code availability

All code for analysis is available upon reasonable request from the corresponding author.

## Declarations

### Employment

DB and MT are employees of Savana, which was a paid consultant to BMS-Pfizer Alliance. DA and MAHP are employees of Pfizer Company, one of the study sponsors.
