# Correction: Prediction model for major bleeding in anticoagulated patients with cancer-associated venous thromboembolism using machine learning and natural language processing

**DOI:** 10.1007/s12094-024-03780-2

**Published:** 2024-11-19

**Authors:** Andrés J. Muñoz Martín, Ramón Lecumberri, Juan Carlos Souto, Berta Obispo, Antonio Sanchez, Jorge Aparicio, Cristina Aguayo, David Gutierrez, Andrés García Palomo, Diego Benavent, Miren Taberna, María Carmen Viñuela-Benéitez, Daniel Arumi, Miguel Ángel Hernández-Presa

**Affiliations:** 1 Medical Oncology Service, Hospital General Universitario Gregorio Marañón Universidad Complutense, Madrid, Spain; 2 Hematology Service, Clínica Universidad de Navarra, Pamplona, Spain; 3 CIBERCV, Carlos III Health Institute, Madrid, Spain; 4 Hematology Department, Santa Creu i Sant Pau Hospital, Barcelona, Spain; 5 Oncology Department, Infanta Leonor Hospital, Madrid, Spain; 6 Oncology Department, Puerta de Hierro Hospital, Madrid, Spain; 7 Oncology Department, Polytechnic and University Hospital of La Fé, Valencia, Spain; 8 Oncology Department, Infanta Sofía Hospital, Madrid, Spain; 9 Oncology Department, Fuenlabrada Hospital, Madrid, Spain; 10 Oncology Department, University Hospital of León, León, Spain; 11 Savana Research, Madrid, Spain; 12 Medicine Department, Facultad de Medicina, Universidad Complutense, Madrid, Spain; 13 Pfizer S.L.U. Medical Department, Madrid, Spain

**Correction: Clinical and Translational Oncology** 10.1007/s12094-024-03586-2

In this article the affiliation details for Author María Carmen Viñuela‑Benéitez were incorrectly given as ‘Medical Oncology Service, Hospital General Universitario Gregorio Marañón Universidad Complutense, Madrid, Spain’ but should have been ‘Medicine Department, Facultad de Medicina, Universidad Complutense, Madrid, Spain’.

The original article has been corrected.

