# Supplementary material for: Prediction model for major bleeding in anticoagulated patients with cancer-associated venous thromboembolism using machine learning and natural language processing
Source: Clin Transl Oncol. 2024 Sep 14;27(4):1816–25. doi: 10.1007/s12094-024-03586-2 (PMC12000191; doi:10.1007/s12094-024-03586-2)
Supplement: Supplementary file 1 — Supplementary file1 (DOCX 975 KB) [file 12094_2024_3586_MOESM1_ESM.docx]

**Supplementary Information**

**Supplemental Methods**

# Participating hospital sites

| **Hospital name** | **City (Spain)** | |
| --- | --- | --- |
| Hospital Universitario Infanta Leonor | | Madrid |
| Hospital Universitari i Politècnic La Fe | | Valencia |
| Hospital Universitario Puerta de Hierro | | Madrid |
| Hospital de la Santa Creu i Sant Pau | | Barcelona |
| Hospital Universitario de León | | León |
| Clínica Universidad de Navarra | | Pamplona |
| Hospital Universitario Infanta Sofía | | Madrid |
| Hospital Universitario Fuenlabrada | | Fuenlabrada, Madrid |
| Hospital Universitario Gregorio Marañón | | Madrid |

# Inclusion and exclusion criteria

The study population comprised all adult cancer patients with a diagnosis of VTE under anticoagulant therapy [low-molecular weight heparin (LMWH) and vitamin K antagonist (VKA)] between January 1st, 2014, and December 31st, 2018. The inclusion and exclusion criteria for this study are specified below.

**Inclusion criteria**

Patients must fulfil all following inclusion criteria to be eligible for inclusion in the study:

- Age ≥18 years at index date
- Diagnosis of symptomatic and/ or incidental VTE (including pulmonary embolism, deep vein thrombosis, visceral thrombosis, and VTE related to a central venous catheter) that occurred within six months before or after cancer diagnosis (including any solid or hematological malignancy, except for non-melanoma cancer of the skin), or six months after any cancer treatment, or in patients with a history of recurrent or metastatic cancer.
- Ongoing treatment with anticoagulant therapy

**Exclusion criteria**

Patients meeting any of the following criteria were excluded from the study:

- Anticoagulation treatment for an indication other than VTE
- Diagnosis of malignancy after 6 months from VTE event.
- Non-metastatic patients with cancer diagnosis (and last cancer treatment) > 6 months before VTE diagnosis
- Symptomatic and/or incidental VTE while having cancer in complete remission for > 2 years.
- Diagnosis of acute leukemia.

**Definitions:**

- Active cancer: Presence of the 'cancer' term, as per the SNOMED CT classification- including any solid or hematological malignancy, except for non-melanoma skin cancer, documented within the EHRs. Diagnosis of symptomatic and/or incidental VTE (including pulmonary embolism [PE], deep vein thrombosis [DVT], visceral thrombosis, and VTE related to a central venous catheter) that occurs within six months before or after cancer diagnosis.
- VTE: Diagnosis of symptomatic and/or incidental VTE (including pulmonary embolism, DVT, visceral thrombosis, and VTE related to a central venous catheter) that occurs within 6 months before or after cancer diagnosis.
- VTE diagnosis: VTE was determined based on the clinician's notation within the EHRs, including clinical diagnosis, ultrasound or any method reported by the clinician.

# Sample size calculations

The minimum sample size required to establish the risk of major bleeding (MB) in anticoagulated cancer patients with VTE was 1,050 patients. This was estimated considering the main objective, namely developing a multiple regression logistic model with 100 predictors with an observed small effect size (0.05), a significance level of 0.05, and a statistical power of 0.9.

Assuming balanced classes, the number of required patients experiencing MB events would have been 525. Considering that 75% of the available data had to be used for training (and 25% for validation), that 4.1-9% of anticoagulant-treated cancer patients with thrombosis experience MB^1,2^, the size of the study population was calculated between 17,073 and 20,588 considering the scenarios with the lowest frequencies.

# Technology and evaluation of performance

All study variables were extracted from patients' EHRs using EHRead®, a proprietary technology that uses natural language processing (NLP) to extract clinical entities and their context from free text. This entailed those conceptual definitions of all study variables were pre-specified and mapped to clinical entities present in SNOMED clinical terms, (which is a systematically organized computer-processable collection of medical terms used in clinical documentation), using SNOMED CT browser. This allowed for the translation of unstructured data from various hospital departments into usable variables to extract. The clinical accuracy of the conceptual definitions and entity mapping was reviewed and approved by two medical research experts specialized in NLP.

To ensure the quality of data extraction, EHRead® performance was externally evaluated comparing the results of the technology in a corpus of medical records in which key entities were annotated by specialists from the participating institutions. After clinical entity extraction, variables were constructed by applying dedicated data wrangling operations to their mapped entities, leveraging specific NLP parameters generated by dedicated ML models (e.g., negation, temporality, attributes, etc.) and record-specific metadata (e.g., date, medical department, record type, etc.).

The evaluation of the performance of *EHRead^®^* involved the following phases:

- Text collection. In NLP systems, the amount of data necessary to capture enough linguistic events to ensure consistent and robust performance metrics is an open question. To tackle this issue, we used Savana’s SampLe Calculator for the Evaluation (SLiCE) calculator. This calculator indicates the minimum number of annotated EHRs required to obtain the expected parameters based on the prevalence in the EHRs of the main study variable (in this case, cancer and VTE). The parameters used for this calculation included a confidence level of 95% (α = 5%), interval widths of 10% (percentage points), and expected values of precision and recall. Thus, SliCE provides a robust estimation of P and R assuring that the true value is at ±5% (pp) with a confidence level of 95%.
- Annotation task. The overall goal of this phase was to evaluate the system’s accuracy when identifying records that contained mentions of cancer and VTE. To build the standard corpus, a set of documents was first pre-annotated using Savana’s *EHRead^®^* technology; these documents included key study variables to identify the population with cancer and VTE. Then, these documents were then corrected manually via Savana’s Evaluation Tool.
- Annotation of the standard. Two designated expert physicians (hereby referred to as ‘the annotators’) at each hospital annotated the set of randomly selected records. Annotators followed the annotation guidelines written by Savana’s medical team. Then, the Inter-Annotator Agreement (IAA) was measured using the F1-Score to ensure the consistency of the guidelines and the reliability of the annotation. The IAA is a metric that indicates the extent to which the different annotators converged in their evaluation, thus providing information regarding the difficulty of the task. Finally, a third physician acted as judge, reviewing the annotations made by the two annotators and resolving any possible discrepancies. The resulting standard corpus served as a resource for the evaluation of the performance of Savana technology.
- Evaluation. The system evaluation was calculated in terms of the standard metrics of Precision, Recall, and their harmonic mean F1-Score
- *Precision* =
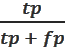
. This parameter indicates the accuracy of the system in retrieving key clinical concepts.
- *Recall* =
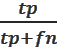
 . This parameter indicates the amount of information the system retrieves.
- *F1-Score* =
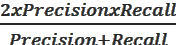
. This parameter gives us an overall performance indicator of information retrieval.

In all cases, *tp* is the number of true positives (i.e., records correctly retrieved), *fn* is the set of false negatives (i.e., records incorrectly not retrieved), and *fp* is the number of false positives (i.e., records incorrectly retrieved).

The results of performance metrics of the technology for this study are shown in Table S1.

# Period prevalence and incidence calculations

In-hospital period prevalence estimations (2014-2018) were calculated according to the formula:

Prevalence = existing cases at midpoint of the study period/hospital population,

in which patients at midpoint were those with cancer and VTE, having available EHRs and no exitus between 6/15/2015 and 6/15/2016.

In-hospital annual incidence estimations were calculated according to the formula:

Incidence = new cases in a given year/hospital population at risk in that year,in which the population at risk comprised patients with cancer and VTE with available EHRs in both the year of interest and previous years. To estimate yearly incidences, only data from sites with available records for the year of interest were analyzed.

Of note, both calculations are in-hospital estimations and were over the entire source population at the midpoint of the study. For clarity, the term 'in-hospital' in this study is employed to denote instances of VTE or bleeding diagnosed during a patient hospital admission, thereby excluding diagnoses made in primary care or other outpatient environments.
MB rates were assessed by dividing the number of patients with MB by the sum of each patient’s observation time. Observation time was the time from index date to MB (or to follow-up end in patients with no MB).

# Predictive model for MB in patients with cancer and VTE

In this study, we aimed to develop a predictive model of MB in patients with active cancer and VTE. To define the model outcome, we selected the date of primary VTE diagnosis as the timepoint in which to perform the prediction (i.e., index). Then we established a time window from index to six months follow-up to determine whether there was an MB event in each patient. Given the binary nature of this outcome, we resorted to develop a classification predictive model. Patients with an MB event within six months after index were considered the positive class, whereas patients with no MB event within six months after index were labeled as the negative class. In accordance, patients with no MB event and less than six months of available follow-up (i.e., missing outcome) were excluded. Patients with previous MB events were also excluded from this analysis.

The predictive analysis pipeline involved the following steps: (1) population selection to train the model, (2) feature selection, and (3) model training and validation.

**Step 1: Population selection**

This step aimed to maximize the information available while removing biases that could lead to a model of limited application. It consisted of three major stages:

1. Selection of the most informative patients: To leverage the vast amount of information available in our records, imputation was used to complete missing data. Though, applying imputation methods for variables with missing values in >20% of patients is not recommended^3^. To avoid excluding some of our most informative variables (e.g., laboratory parameters, which showed missing values above 20%) we resorted to excluding the least informative patients instead. We used the custom Information Score algorithm described below.
2. Data imputation: We applied random forest multiple imputation (MissForest R package) to variables with missing data in <20% of patients.
3. Data down-sampling: When there is an outcome class imbalance, predictive models learn mainly from examples of the majority class (i.e., patients with no VTE recurrence). This could lead to a model that fails to correctly predict VTE recurrence. To prevent this, we resorted to down-sample the negative class. We selected a random sample of patients with no VTE recurrence to match the patients with VTE recurrence and achieve a 1:1 class ratio.

**Step 2: Feature selection**

This step was used to identify the most informative, high-quality, and non-redundant variables to predict VTE recurrence. Our initial set comprised a set of 113 variables with over 10 possible interactions. To reduce this complexity and prevent overfitting, we combined some basic selection steps (e.g., clinically-driven selection) with ML techniques, which are known to efficiently large explore feature spaces. This procedure consisted of the following stages:

1. Manual elimination of variables with redundant information: Some variables included in our initial set presented clinical interdependence (e.g., hemoglobin and anemia). We pre-selected variables, favoring those expected to contain more information (i.e., numerical variables).
2. Elimination of invariant variables (with zero variance).
3. Elimination of variables that had > 20% missing values, even after the application of the Information Score algorithm.
4. Elimination of highly (multi) collinear variables: Variables with a variance inflation factor (VIF) > 5 in a (multi) collinearity analysis were eliminated.
5. Random forest feature selection: 500 random forest models were trained using all the remaining variables as predictors. Then, the variables that appeared in <5% of the models with an importance (mean decrease accuracy) >0.05 were excluded due to their low predictive potential.

**Step 3: Model training and validation**

Three types of models (i.e., logistic regression, decision tree, and random forest, from the Scikit learn Python package) were trained and evaluated using the model dataset generated in Step 1, and they all considered the predictor variables resulting from Step 2.

This step proceeded as follows:

1. Data was randomly split into a train set (75% of the data) and test set (remainder 25%).
2. All three models were trained using the train set data.
3. The resulting models were internally validated using the test set data. Performance was measured by confusion matrix metrics and by ROC (Figure S2) and calibration curves (Figure S3). Confidence intervals (CI) were estimated by bootstrapping with 1000 splits with resampling.
4. Models were compared using their performance metrics (prioritizing receiver operating characteristic - area under the curve [ROC-AUC]). Statistical significance was considered when CIs were non-overlapping.

## Information Score algorithm

In order to leverage the data collected on numeric variables with high number of missing values (i.e., >20%), we introduced the Information Score (InfoScore) algorithm. This algorithm allowed to utilize these variables by keeping patients with higher levels of information and completing data using randomly selected patients with less information until the variables of interest presented a ≤20% of missing data. The steps involved in the development of the algorithm are described below:

1. Computation of InfoScore obtaining the proportion of missing values for each patient.
2. Creation of an output dataset consisting of patients with InfoScore >= 0.8.
3. Randomly selection of a patient with InfoScore <0.8 adding it to the output dataset. Repetition of this step iteratively until one variable in the output dataset reaches 20% of missing values.
4. Return of the output dataset with the selected patients.

For the Information Score selection, an initial threshold to split the population was defined. We empirically selected a threshold of 0.8. From there, the remaining steps were performed over the data considering a pre-selected set of numeric variables. Variables were selected considering their number of missing values. The more missing values, the more constrained the final dataset. Thus, variables with >50% of missing values were not considered for the InfoScore patient selection, as they would heavily stretch the population.

# External validation of the CAT-BLEED score

We resorted to validating the CAT-BLEED score^4^ using our data, to assess whether our newly developed MB predictive model for cancer patients with VTE and AC treatment outperformed previously developed tools:

1. Outcome definition: the outcome of CAT-BLEED was “clinically relevant bleeding within 6 months of VTE”, which involves the union of MB and CRNMB. Hence, for this validation, we modified the definition of the outcome described in the previous section to also include CRNMB.
2. Predictor definition: we engineered the predictors from CAT-BLEED in our data set:

- Age >75 years: whether age at index was > 75 years or not.
- Regionally advanced or metastatic cancer: union of metastasis, stage III, and stage IV detections in the patients’ EHRs before index or at index.
- Genitourinary tumor: detection of affirmed prostate, kidney, ureter, bladder, urethra, or genitourinary cancer in the patients’ EHRs before index or at index.
- Gastrointestinal cancer and edoxaban treatment: intersection of affirmed edoxaban and gastrointestinal cancer (union of gastrointestinal, esophageal, gastric, pancreatic, liver, biliary duct, gallbladder, small intestine, large intestine, colorectal cancer or amulloma) detections in the patient’s EHRs before index or at index.
- Anticancer therapies considered to be associated with gastrointestinal toxicity in the past 4 weeks: detection in the patient’s EHRs of any treatment of the following groups between 4 weeks before index and index: Alkylating agents, Antimetabolites, Antimitotic agents, Epidermal growth factor receptor inhibitors, Immunomodulating agents, Mitogen-activated protein kinase inhibitors, Nitrosourea, Phosphatidylinositol-4,5-bisphosphate 3-kinase inhibitors, Topoisomerase inhibitors, Vascular endothelial growth factor (receptor) inhibitors.
- Creatinine clearance (ml/min): the closest value to index (with a window of -6 to 0 months) of serum creatinine (mg/dl) detections was selected from the patients’ EHRs. Then, the estimated glomerular filtration rate (eGFR) was inferred from creatinine levels, age, and sex using the modified CKD-EPI creatinine equation without race^5^. Then, body surface area (BSA) (m^2^) was calculated using the Mosteller formula: √[(height in cm × weight in kg) / 3600]. For patients with no height or weight information, BSA was imputed as 1.73 m². Finally, creatinine clearance was calculated by multiplying eGFR with BSA.

1. Score calculation: we applied the CAT-BLEED score equation^4^ to our data set. We used the test set defined in the previous section to allow comparisons with the newly developed models. According to the authors, patients with a score > 14.2% are at medium or high risk of developing MB. Using this threshold, we translated the score into a binary classification prediction.
2. Model evaluation: model performance and comparison were performed as described in the previous section.

# Supplemental Results

**1. Supplemental Tables**

Table S1 Performance of EHRead® identifying records that contained key study variables.

| **Variable** | **Sensitivity** | **PPV** | **F1-Score** |
| --- | --- | --- | --- |
| Anemia | 0.93 | 0.81 | 0.86 |
| Bemiparin | 0.95 | 0.94 | 0.95 |
| Brain cancer | 0.65 | 0.64 | 0.65 |
| Brain metastasis | 0.70 | 1 | 0.82 |
| Chemotherapy | 0.95 | 0.90 | 0.92 |
| Deep vein thrombosis | 0.75 | 0.91 | 0.82 |
| Enoxaparin | 0.95 | 0.72 | 0.82 |
| Gastric cancer | 0.72 | 0.98 | 0.83 |
| Hormone therapy | 0.87 | 0.92 | 0.89 |
| Intra-articular hemorrhage | 0.85 | 0.82 | 0.83 |
| Intracranial hemorrhage | 0.77 | 0.75 | 0.76 |
| Lung cancer | 0.79 | 0.75 | 0.77 |
| Pancreatic cancer | 0.79 | 0.66 | 0.72 |
| Portal thrombosis | 0.92 | 0.96 | 0.94 |
| Pulmonary embolism | 0.90 | 0.82 | 0.86 |
| Radiotherapy | 0.88 | 0.88 | 0.88 |
| Splenic thrombosis | 0.83 | 0.97 | 0.89 |
| Thrombopenia | 0.86 | 0.88 | 0.87 |
| Venous thromboembolism | 0.60 | 0.76 | 0.66 |
| PPV: positive predictive value. | | | |

Table S2 Pharmacological treatments at baseline

|  | **N (%)**  **21227 (100)** |
| --- | --- |
| ***Anticoagulant treatment n (%)*** |  |
| Low-molecular-weight heparin (LMWH) | 17,830 (84) |
| Vitamin-K antagonists (VKA) | 4,710 (22.2) |
| LMWH and VKA* | 3,414 (16) |
| ***Antineoplastic agents n (%)*** |  |
| Platinum compounds | 1747 (8.2) |
| Carboplatin | 758 (3.6) |
| Cisplatin | 697 (3.3) |
| Oxaliplatin | 437 (2.1) |
| Taxanes | 1088 (5.1) |
| Paclitaxel | 769 (3.6) |
| Docetaxel | 353 (1.7) |
| Cabazitaxel | 33 (0.2) |
| Pyrimidine analogues | 941 (4.4) |
| Gemcitabine | 574 (2.7) |
| Fluorouracil | 338 (1.6) |
| Cytarabine | 40 (0.2) |
| Tegafur | 12 (0.1) |
| Azacitidine | 11 (0.1) |
| Folic acid analogues | 737 (3.5) |
| Methotrexate | 448 (2.1) |
| Pemetrexed | 272 (1.3) |
| Raltitrexed | 26 (0.1) |
| Nitrogen mustard analogues | 511 (2.4) |
| Cyclophosphamide | 330 (1.6) |
| Ifosfamide | 99 (0.5) |
| Melphalan | 73 (0.3) |
| Bendamustine | 38 (0.2) |
| Vinca alkaloids | 287 (1.4) |
| Vinorelbine | 167 (0.8) |
| Vincristine | 87 (0.4) |
| Vinblastine | 20 (0.1) |
| Vinflunine | 14 (0.1) |
| Anthracyclines and related substances | 233 (1.1) |
| Doxorubicin | 177 (0.8) |
| Epirubicin | 46 (0.2) |
| Derivatives of podophyllotoxin | 220 (1) |
| Etoposide | 220 (1) |
| Purine analogues | 54 (0.3) |
| Thioguanine | 31 (0.1) |
| Fludarabine | 20 (0.1) |
| Other antineoplastic agents | 261 (1.2) |
| Irinotecan | 193 (0.9) |
| Bortezomib | 146 (0.7) |
| Topotecan | 38 (0.2) |
| Tretinoin | 23 (0.1) |
| ***Monoclonal antibodies n (%)*** |  |
| Panitumumab | 790 (3.7) |
| Bevacizumab | 461 (2.2) |
| Rituximab | 219 (1) |
| Immunotherapy | 218 (1) |
| Cetuximab | 173 (0.8) |
| Trastuzumab | 127 (0.6) |
| Pertuzumab | 24 (0.1) |
| ***Tyrosine Kinase Inhibitors n (%)*** |  |
| Sorafenib | 86 (0.4) |
| Erlotinib | 77 (0.4) |
| Regorafenib | 67 (0.3) |
| Sunitinib | 64 (0.3) |
| Gefitinib | 50 (0.2) |
| Pazopanib | 48 (0.2) |
| Imatinib | 27 (0.1) |
| Afatinib | 25 (0.1) |
| Crizotinib | 23 (0.1) |
| Axitinib | 16 (0.1) |
| Dabrafenib | 12 (0.1) |
| Lapatinib | 12 (0.1) |
| Treatments classified per ATC4-ATC5. Data extracted using a temporal window of 6 months around index date. * Administration of LMWH and VKA in the time range analyzed, not necessarily simultaneously. | |

**Table S3 Laboratory results at baseline**

|  | N = 21227 |
| --- | --- |
| *Hematology* |  |
| Hemoglobin (g/dl) |  |
| N (%) | 18884 (89) |
| Mean (SD) | 11.75 (2.48) |
| Median (Q1, Q3) | 11.8 (10.2, 13.4) |
| Platelet count (10^3^/mm^3^) |  |
| N (%) | 17110 (80.6) |
| Mean (SD) | 244.11 (128.16) |
| Median (Q1, Q3) | 224 (165, 296) |
| Leukocyte count (10^3^/mm^3^) |  |
| N (%) | 16120 (75.9) |
| Mean (SD) | 13.88 (51.54) |
| Median (Q1, Q3) | 8.37 (6.1, 11.56) |
| Leukocytosis n (%)^1^ | 8527 (40.2) |
| Thrombocytosis n (%)^2^ | 4122 (19.4) |
| Anemia n (%)^3^ | 12997 (61.2) |
| *Biochemistry* |  |
| Serum creatinine (mg/dl) |  |
| N (%) | 17071 (80.4) |
| Mean (SD) | 1.26 (2.56) |
| Median (Q1, Q3) | 0.88 (0.69, 1.2) |
| Total bilirubin (mg/dl) |  |
| N (%) | 10717 (50.5) |
| Mean (SD) | 1.08 (2.53) |
| Median (Q1, Q3) | 0.52 (0.36, 0.9) |
| *Coagulation values* |  |
| International normalized ratio (INR) |  |
| N (%) | 15175 (71.5) |
| Mean (SD) | 1.3 (0.9) |
| Median (Q1, Q3) | 1.1 (1.01, 1.25) |
| Data extracted using a temporal window of ± 6 months around index date.^1^ Leukocytosis identified by presence of the term ‘leukocytosis’ in the EHR or leukocytes >12000/mm^3^; ^2^ Thrombocytosis identified by presence of the term ‘thrombocytosis’ in the EHR or platelets > 400000/mm; ^3^ Anemia identified by presence of the term ‘anemia’ in the EHR or hemoglobin <11g/dL. | |

**Table S4 Incidence of MB and CRNMB during follow-up by cancer type.**

|  | Overall  (21227) | Prostate cancer  (1191) | Lung cancer  (1810) | Colorectal cancer  (2143) | Gastric cancer  (423) | Pancreatic cancer  (1054) | Bladder cancer  (1502) |
| --- | --- | --- | --- | --- | --- | --- | --- |
| Major bleeding events  (ISTH criteria) n (%) | 3497 (16.5) | 179 (15) | 357 (19.7) | 334 (15.6) | 70 (16.5) | 197 (18.7) | 269 (17.9) |
| CRNMB  (ISTH criteria) n (%) | 3457 (16.3) | 260 (21.8) | 310 (17.1) | 339 (15.8) | 69 (16.3) | 156 (14.8) | 360 (24) |
| Both n (%) | 969 (4.6) | 62 (5.2) | 91 (5) | 92 (4.3) | 21 (5) | 48 (4.6) | 101 (6.7) |
| None n (%) | 15242 (71.8) | 814 (68.3) | 1234 (68.2) | 1562 (72.9) | 305 (72.1) | 749 (71.1) | 974 (64.8) |
|  | Breast cancer  (1395) | Cervix cancer  (570) | Endometrial cancer  (818) | | Ovarian cancer  (187) | Vaginal cancer  (2) | Vulvar cancer  (12) |
| Major bleeding events  (ISTH criteria) n (%) | 196 (14.1) | 89 (15.6) | 127 (15.5) | | 33 (17.6) | 1 (50) | 2 (16.7) |
| CRNMB  (ISTH criteria) n (%) | 151 (10.8) | 96 (16.8) | 131 (16) | | 37 (19.8) | 1 (50) | 3 (25) |
| Both n (%) | 39 (2.8) | 21 (3.7) | 36 (4.4) | | 13 (7) | 1 (50) | 1 (8.3) |
| None n (%) | 1087 (77.9) | 406 (71.2) | 596 (72.9) | | 130 (69.5) | 1 (50) | 8 (66.7) |
|  | Kidney cancer  (299) | Brain cancer  (421) | Esophageal cancer (376) | Thyroid cancer  (155) | Hematological cancer (1548) | Bile duct cancer  (222) | Tumors with mucosal involvement (2321) |
| Major bleeding events  (ISTH criteria) n (%) | 57 (19.1) | 101 (24) | 75 (19.9) | 18 (11.6) | 243 (15.7) | 45 (20.3) | 368 (15.9) |
| CRNMB  (ISTH criteria) n (%) | 63 (21.1) | 50 (11.9) | 62 (16.5) | 23 (14.8) | 242 (15.6) | 28 (12.6) | 373 (16.1) |
| Both n (%) | 17 (5.7) | 14 (3.3) | 19 (5.1) | 3 (1.9) | 72 (4.7) | 10 (4.5) | 99 (4.3) |
| None n (%) | 196 (65.6) | 284 (67.5) | 258 (68.6) | 117 (75.5) | 1135 (73.3) | 159 (71.6) | 1679 (72.3) |
| Data extracted from index date to last datapoint available during follow-up. CRNMB: clinically relevant non-major bleeding; ISTH: International Society on Thrombosis and Haemostasis | | | | | |  |  |

**Table S5 Initial set of variables (predictors) considered for the development of the predictive model for MB and reasons for exclusion**

| ***Variable #*** | ***Variable name*** | ***Reason for exclusion from the predictive pipeline*** |
| --- | --- | --- |
| 1 | Female sex | Low predictive potential |
| 2 | Age | Included as predictor in MB model |
| 3 | Pulmonary embolism | Low predictive potential |
| 4 | Deep vein thrombosis | Low predictive potential |
| 5 | Synchronic pulmonary and deep vein thrombosis | Collinearity |
| 6 | Visceral vein thrombosis | Low predictive potential |
| 7 | Splenic thrombosis | Grouped in category |
| 8 | Portal thrombosis | Grouped in category |
| 9 | Colorectal cancer | Low predictive potential |
| 10 | Lung cancer | Low predictive potential |
| 11 | Hematologic cancer | Low predictive potential |
| 12 | Bladder cancer | Low predictive potential |
| 13 | Breast cancer | Low predictive potential |
| 14 | Prostate cancer | Low predictive potential |
| 15 | Pancreatic cancer | Low predictive potential |
| 16 | Endometrial cancer | Low predictive potential |
| 17 | Cervix cancer | Low predictive potential |
| 18 | Gastric cancer | Low predictive potential |
| 19 | Brain cancer | Low predictive potential |
| 20 | Esophageal cancer | Low predictive potential |
| 21 | Kidney cancer | Low predictive potential |
| 22 | Bile duct cancer | Low predictive potential |
| 23 | Ovarian cancer | Low predictive potential |
| 24 | Thyroid cancer | Low predictive potential |
| 25 | Vulvar cancer | Low predictive potential |
| 26 | Vaginal cancer | Low predictive potential |
| 27 | Complete remission | Low predictive potential |
| 28 | Metastatic | Included as predictor in MB model |
| 29 | Adenocarcinoma | Low predictive potential |
| 30 | Tumors with mucosal involvement | Collinearity |
| 31 | Alcohol consumption | Missing values |
| 32 | Smoking | Missing values |
| 33 | Venous thromboembolism (family) | Low predictive potential |
| 34 | Hypertension | Low predictive potential |
| 35 | Diabetes mellitus | Low predictive potential |
| 36 | Dyslipidemia | Low predictive potential |
| 37 | Atrial fibrillation | Low predictive potential |
| 38 | Heart failure | Low predictive potential |
| 39 | COPD | Low predictive potential |
| 40 | Asthma | Low predictive potential |
| 41 | Kidney failure | Low predictive potential |
| 42 | Chronic hepatopathy | Low predictive potential |
| 43 | Psychiatric disorders | Low predictive potential |
| 44 | Peripheral artery disease | Low predictive potential |
| 45 | Neurological diseases | Low predictive potential |
| 46 | Varicose veins | Low predictive potential |
| 47 | Acute myocardial infarction | Low predictive potential |
| 48 | Cerebrovascular accident (CVA) | Low predictive potential |
| 49 | Transient ischemic attack (TIA) | Low predictive potential |
| 50 | Inherited thrombophilia | Low predictive potential |
| 51 | Central venous catheter | Low predictive potential |
| 52 | Major bleeding events (ISTH criteria) | Outcome in MB model |
| 53 | CRNMB (ISTH criteria) | Low predictive potential |
| 54 | Vitamin K antagonists | Low predictive potential |
| 55 | Low-molecular weight heparin (LMWH) | Low predictive potential |
| 56 | Acetyl salicylic acid | Low predictive potential |
| 57 | Clopidogrel | Low predictive potential |
| 58 | Ticlopidine | Low predictive potential |
| 59 | Dipyridamole | Low predictive potential |
| 60 | Triflusal | Low predictive potential |
| 61 | Prasugrel | Low predictive potential |
| 62 | Cilostazol | Low predictive potential |
| 63 | Ticagrelor | Low predictive potential |
| 64 | Ditazole | Invariant |
| 65 | Calcium carbasalate | Invariant |
| 66 | Epoprostenol | Low predictive potential |
| 67 | Indobufen | Invariant |
| 68 | Iloprost | Low predictive potential |
| 69 | Abciximab | Low predictive potential |
| 70 | Eptifibatide | Invariant |
| 71 | Nitrogen mustard analogues | Low predictive potential |
| 72 | Purine analogues | Low predictive potential |
| 73 | Pyrimidine analogues | Low predictive potential |
| 74 | Taxanes | Low predictive potential |
| 75 | Folic acid analogues | Low predictive potential |
| 76 | Vinca alkaloids | Low predictive potential |
| 77 | Platinum compounds | Low predictive potential |
| 78 | Anthracyclines and related substances | Low predictive potential |
| 79 | Tretinoin | Grouped in category |
| 80 | Topotecan | Grouped in category |
| 81 | Irinotecan | Grouped in category |
| 82 | Bortezomib | Grouped in category |
| 83 | Bevacizumab | Low predictive potential |
| 84 | Trastuzumab | Low predictive potential |
| 85 | Pertuzumab | Low predictive potential |
| 86 | Rituximab | Low predictive potential |
| 87 | Cetuximab | Low predictive potential |
| 88 | Panitumumab | Low predictive potential |
| 89 | Immunotherapy | Low predictive potential |
| 90 | Tyrosine Kinase Inhibitors | Low predictive potential |
| 91 | Imatinib | Grouped in category |
| 92 | Gefitinib | Grouped in category |
| 93 | Erlotinib | Grouped in category |
| 94 | Sunitinib | Grouped in category |
| 95 | Sorafenib | Grouped in category |
| 96 | Lapatinib | Grouped in category |
| 97 | Pazopanib | Grouped in category |
| 98 | Afatinib | Grouped in category |
| 99 | Crizotinib | Grouped in category |
| 100 | Axitinib | Grouped in category |
| 101 | Regorafenib | Grouped in category |
| 102 | Dabrafenib | Grouped in category |
| 103 | Hemoglobin (g/dl) | Included as predictor in MB model |
| 104 | Platelet count (103/mm3) | Included as predictor in MB model |
| 105 | Leukocyte count (103/mm3) | Included as predictor in MB model |
| 106 | Leukocytosis | Low predictive potential |
| 107 | Thrombocytosis | Low predictive potential |
| 108 | Anemia | Collinearity |
| 109 | Serum creatinine (mg/dl) | Included as predictor in MB model |
| 110 | C-reactive protein (CRP) (mg/l) | Missing values |
| 111 | Total bilirubin (mg/dl) | Missing values |
| 112 | International normalized ratio (INR) | Missing values |
| 113 | Prothrombin activation products | Missing values |

**Table S6 Descriptive data in the train vs. test patient sets following data transformations**

| Variable | Train (N = 1348) | Test (N = 450) |
| --- | --- | --- |
| Major bleeding (output) (%)* | 50.52% | 48.44% |
| *Hemoglobin (g/dl)* |  |  |
| Median (Q1, Q3) | 12.1 (10.2, 13.45) | 12 (10.2, 13.4) |
| *Serum creatinine (mg/dl)* |  |  |
| Median (Q1, Q3) | 0.9 (0.72, 1.22) | 0.9 (0.71, 1.2) |
| *Platelet count (10^3^/mm^3^)* |  |  |
| Median (Q1, Q3) | 220.12 (170, 278) | 218.22 (170.12, 280) |
| *Leukocyte count (10^3^/mm^3^)* |  |  |
| Median (Q1, Q3) | 8.6 (6.41, 11.55) | 8.4 (6.24, 12.12) |
| *Age* |  |  |
| Median (Q1, Q3) | 69 (59, 79) | 69 (58, 80) |
| *Metastasis (%)* | 50.45% | 52.67% |

* Major bleeding rates in the train and test patient sets considered in the predictive model result from the application of down sampling techniques to balance the classes in this variable. See descriptive data in Table S5 for MB rates in the study population during follow-up.

**Table S7 Model performance metrics**

| **Metric** | **LR** | **DT** | **RF** | **CAT-BLEED** |
| --- | --- | --- | --- | --- |
| TN | 120 (105, 135) | 122 (108, 138) | 129 (114, 144) | 156 (143, 168) |
| TP | 141 (127, 155) | 145 (131, 158) | 136 (122, 150) | 82 (67, 97) |
| FN | 77 (63, 91) | 73 (60, 87) | 82 (68, 96) | 152 (137, 167) |
| FP | 112 (97, 127) | 110 (94, 124) | 103 (88, 118) | 60 (48, 73) |
| ROC-AUC | 0.603 (0.552, 0.654) | 0.599 (0.547, 0.648) | 0.612 (0.562, 0.661) | 0.534 (0.485, 0.587) |
| Accuracy | 0.580 (0.538, 0.624) | 0.593 (0.549, 0.638) | 0.589 (0.544, 0.633) | 0.529 (0.487, 0.569) |
| Precision | 0.556 (0.519, 0.598) | 0.568 (0.529, 0.611) | 0.568 (0.528, 0.613) | 0.577 (0.510, 0.639) |
| Recall | 0.647 (0.583, 0.711) | 0.665 (0.601, 0.725) | 0.624 (0.56, 0.688) | 0.350 (0.286, 0.415) |
| F1-score | 0.598 (0.553, 0.644) | 0.613 (0.568, 0.654) | 0.595 (0.548, 0.639) | 0.436 (0.371, 0.493) |

Data expressed as median, 95% CI. LR: Logistic Regression; DT: Decision Tree; RF: Random Forest; TN: True Negative; TP: True Positive; FN: False Negative; FP: False Positive

**2.** **Supplemental Figures**

**Fig. S1**


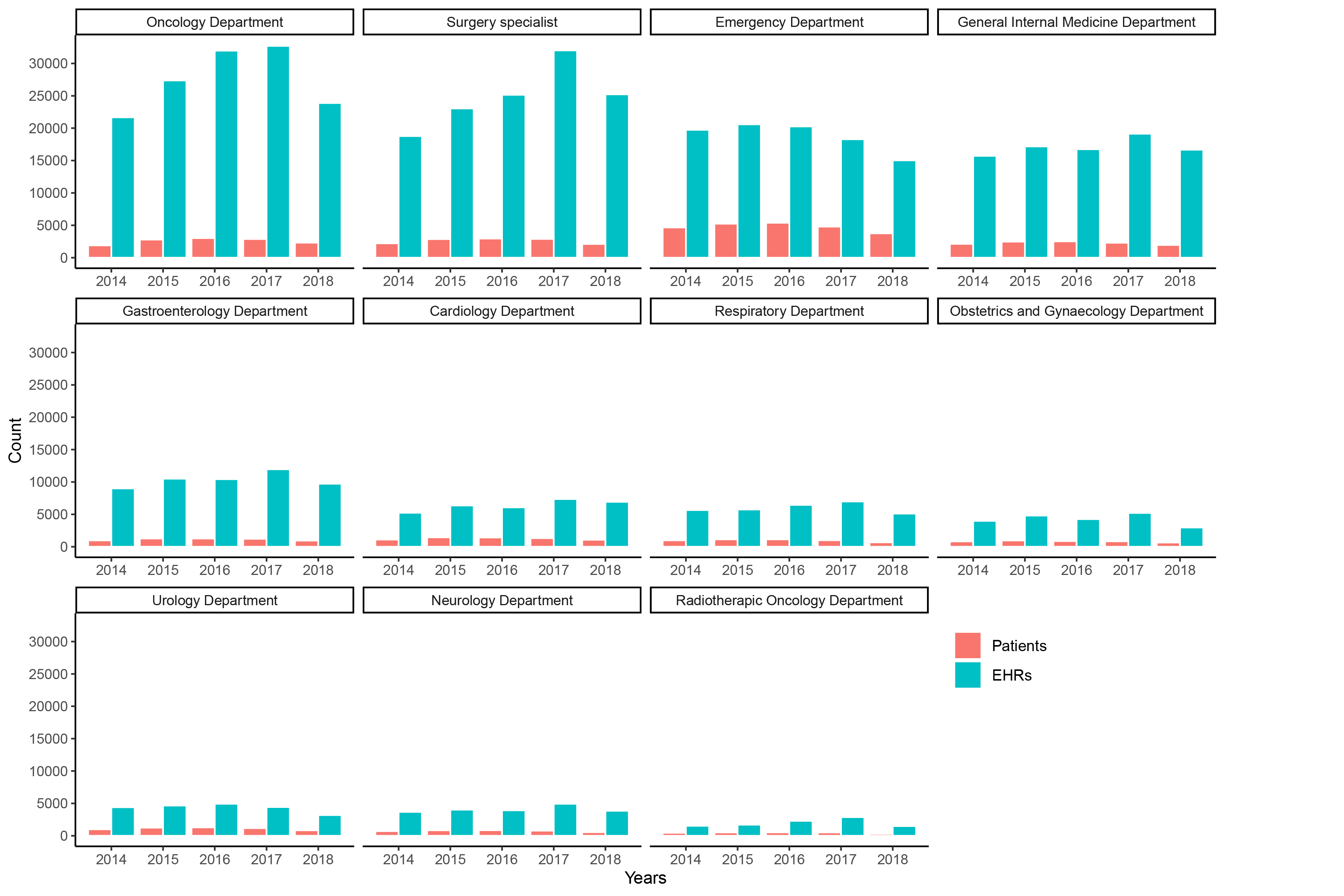


Figure S1. Number of patients and electronic health records (EHRs) per hospital Department across the study period. Bar plots show patient and EHR counts per hospital/year of study period. Plots are sorted by total number of EHRs per Department across the whole study period. EHRs that do not contain mention of the study variables were excluded

**Fig. S2
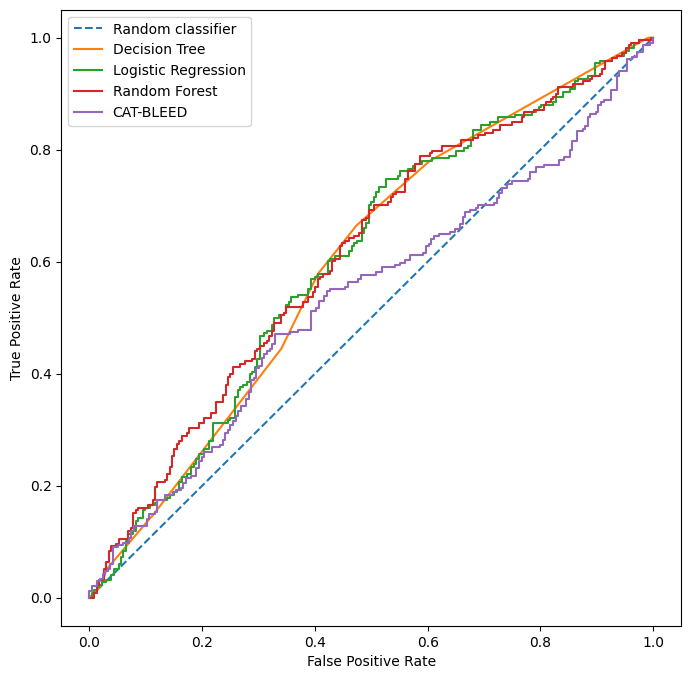
**

Figure S2. Receiver operating characteristic (ROC) curves for the generated predictive models for major bleeding. ROC curves for the decision tree model (AUC-ROC = 0.60) (yellow line), logistic regression model (AUC-ROC = 0.60) (green line), and random forest model (AUC-ROC =0.61) (red line). The dashed blue line represents random performance

**Fig. S3**

**
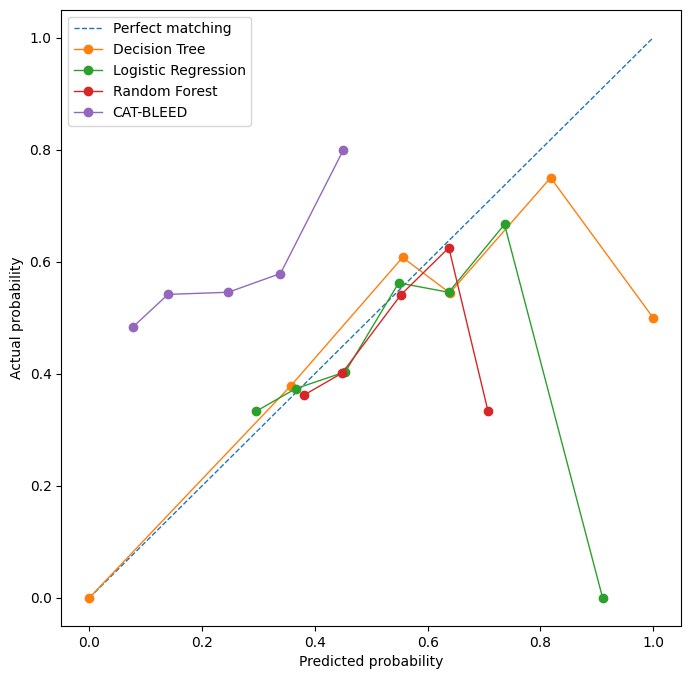
**

**Figure S3.** Calibration curves for the generated predictive models for major bleeding in the validation set. Calibration curves for logistic regression model (green line), the decision tree model (yellow line), and random forest model (red line). The dashed blue line represents maximum calibration

**Fig. S4**
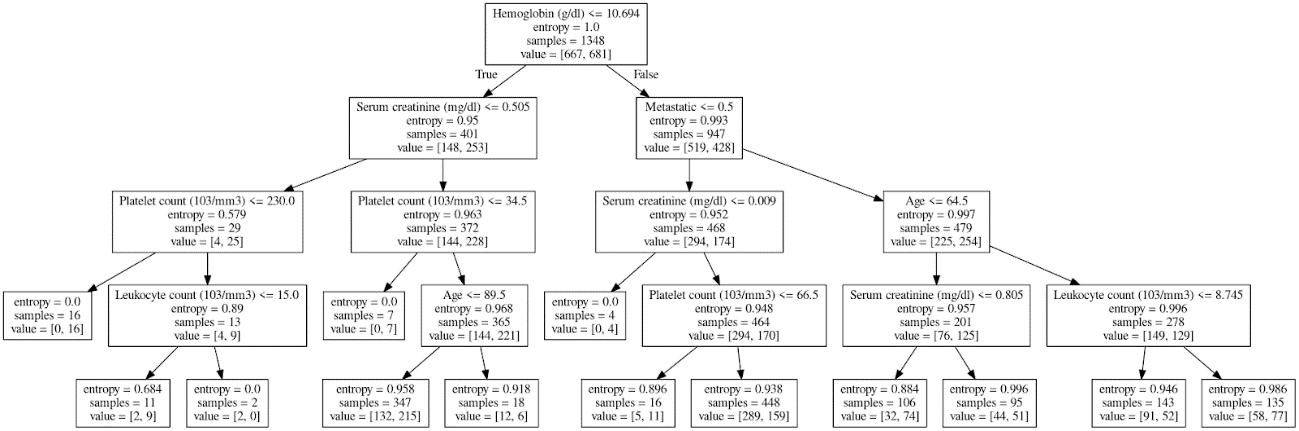


Figure S4. Decision tree model for major bleeding events

# References

1 Trujillo-Santos, J. *et al.* Predicting recurrences or major bleeding in cancer patients with venous thromboembolism. Findings from the RIETE Registry. *Thrombosis and haemostasis* **100**, 435-439 (2008).

2 Chee, C. E. *et al.* Predictors of venous thromboembolism recurrence and bleeding among active cancer patients: a population-based cohort study. *Blood* **123**, 3972-3978, doi:10.1182/blood-2014-01-549733 (2014).

3 Kokla, M., Virtanen, J., Kolehmainen, M., Paananen, J. & Hanhineva, K. Random forest-based imputation outperforms other methods for imputing LC-MS metabolomics data: a comparative study. *BMC Bioinformatics* **20**, 492, doi:10.1186/s12859-019-3110-0 (2019).

4 de Winter, M. A. *et al.* Estimating Bleeding Risk in Patients with Cancer-Associated Thrombosis: Evaluation of Existing Risk Scores and Development of a New Risk Score. *Thrombosis and haemostasis*, doi:10.1055/s-0041-1735251 (2021).

5 Inker, L. A. *et al.* New Creatinine- and Cystatin C-Based Equations to Estimate GFR without Race. *N Engl J Med* **385**, 1737-1749, doi:10.1056/NEJMoa2102953 (2021).
